# Supplementary material for: Conditional chemoconnectomics (cCCTomics) as a strategy for efficient and conditional targeting of chemical transmission
Source: eLife. 2024 Apr 30;12:RP91927. doi: 10.7554/eLife.91927 (PMC11060718; doi:10.7554/eLife.91927)
Supplement: Figure 3—source data 3. [file elife-91927-fig3-data3.zip › Figure 3 source data3 part 1.zip!/Figure 3-Source data 3-part1/1071-1-CRISPResso_Report_osfbjvga.zip!/CRISPResso_on_1071_2/2b.Nucleotide_percentage_quilt_around_sgRNA_gaccgtctcaccgtcaagat.pdf]

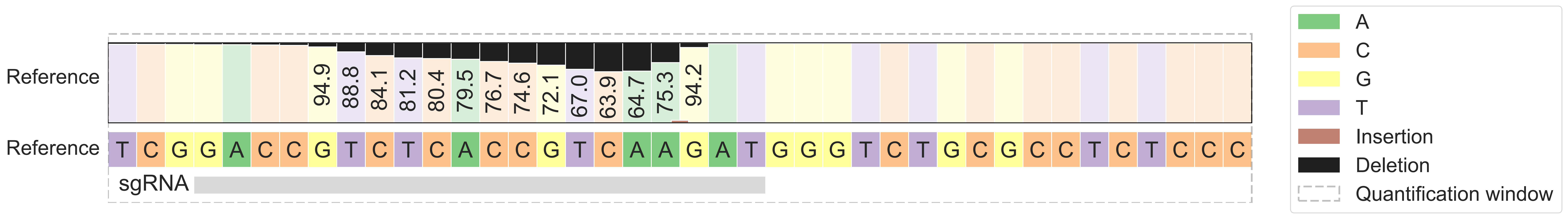
Reference
94.9
88.8
84.1
81.2
80.4
79.5
76.7
74.6
72.1
67.0
63.9
64.7
75.3
94.2
Reference
T C G G A C C G T C T C A C C G T C A A G A T G G G T C T G C G C C T C T C C C
sgRNA
A
C
G
T
Insertion
Deletion
Quantification window
